# Anti-inflammatory properties of raw honey and its clinical applications in daily practice

**DOI:** 10.5339/qmj.2022.fqac.27

**Published:** 2022-04-06

**Authors:** Hashim Mohammed

**Affiliations:** ^1^Family Medicine, Primary Health Care Corporation, Doha, Qatar E-mail: hasmohamed@phcc.gov.qa

**Keywords:** anti-inflammatory, atopic dermatitis, raw honey

## Abstract

Background: Honey is a natural product; and in terms of phytochemical composition, it contains a high number of flavonoids and polyphenols such as quercetin, kaempferol, chrysin, and apigenin. Flavonoids found in honey mitigate the inflammatory processes, thereby exhibiting the anti-inflammatory potential of honey. Besides their anti-oxidant activities, flavonoids have the ability to inhibit pro-inflammatory enzymes such as LOX, COX, iNOS, and pro-inflammatory mediators, including cytokines, nitric oxide, and chemokines. Furthermore, flavonoids modulate transcriptional factors such as NF-?B, thereby controlling the expression of several inflammatory mediators.

Methods: Clinical uses of natural raw honey have been studied at our Umm Ghuwailina Health Center, Doha, Qatar, with daily application under occlusion with promising results, in various patients with multiple conditions, including eczema, seborrheic dermatitis, and psoriasis. A layer of honey was applied to the lesion site, covered with a transparent securement dressing, and washed off for 7 consecutive days for patients with atopic dermatitis and psoriasis. The Three Item Severity (TIS) score, which includes erythema, edema/papulation, and excoriation on a scale from 0 to 3, was used for atopic dermatitis. For psoriasis, the primary outcome measure was the intensity component of the validated Psoriasis Area and Severity Index (PASI) and the Visual Analogue Score (VAS). Participants who were allergic to honey or receiving any corticosteroid within the last one month were excluded.

Results: Twelve patients with atopic dermatitis had a significant improvement from a TIS score of 6 to 1 noticed in the honey-treated lesion 1 week later. With regard to psoriatic lesions, 6 patients were studied. There was a significant overall improvement in their psoriatic lesions, reaching a PASI score of 80 even one month after stopping the topical honey application. The vast majority of patients showed a significant improvement and no side effects.

Conclusion: Raw natural honey might be a promising efficacious and cost-effective alternative to advanced anti-inflammatory products. However, well-designed double-blind placebo-controlled clinical trials are needed to confirm the benefits of honey from different botanical sources.

